# *Angelica sinensis* as a Multi-Targeted Natural Product Candidate: Constituent-Specific Mechanisms, Exposure Constraints, and Translational Development Challenges

**DOI:** 10.3390/ph19071073

**Published:** 2026-07-12

**Authors:** Jialian Song, Hui Sun, Zhineng Li, Guangli Yan, Ling Kong, Lei Liu, Xijun Wang

**Affiliations:** 1 State Key Laboratory of Integration and Innovation of Classic Formula and Modern Chinese Medicine, National Chinmedomics Research Center, Metabolomics Laboratory, Department of Pharmaceutical Analysis, Heilongjiang University of Chinese Medicine, Heping Road 24, Harbin 150040, China; 15193085176@163.com (J.S.); sunhui7045@163.com (H.S.); muhua078@163.com (Z.L.); 15244624557@163.com (L.K.); liulei7711@126.com (L.L.); 2 State Key Laboratory of Quality Research in Chinese Medicine, Macau University of Science and Technology, Avenida Wai Long, Taipa, Macau, China

**Keywords:** *Angelica sinensis*, ligustilide, ferulic acid, polysaccharides, pharmacokinetics, translational pharmacology

## Abstract

*Angelica sinensis* (Oliv.) Diels (*A. sinensis*), commonly known as Danggui, is a chemically complex medicinal plant widely used in East Asian medicine and reported to exert anti-inflammatory, antioxidant, immunomodulatory, hematopoietic, neuroprotective, vasoprotective, metabolic, and tissue-repair effects. This narrative translational review evaluates literature published up to May 2026 and reappraises *A. sinensis* through a constituent-specific and exposure-relevant framework, focusing on phthalides, ferulic acid-related phenolic acids, polysaccharides, and representative preparations. Phthalides are mainly associated with neurovascular protection, mitochondrial homeostasis, autophagy/mitophagy regulation, and anti-apoptotic responses, but their translational relevance is constrained by chemical instability and low oral exposure. Ferulic acid and related phenolic acids are more consistently linked to inflammatory attenuation, redox regulation, epithelial barrier protection, and metabolic stress responses; however, many in vitro studies use concentrations exceeding plausible free systemic exposure. Polysaccharides are most strongly associated with hematopoietic support, immune modulation, gut microbiota regulation, metabolic homeostasis, and microenvironmental remodeling, although structural heterogeneity limits cross-study comparability. Direct clinical evidence for single-herb *A. sinensis* remains limited, and most clinical signals derive from multi-herb formulas. Overall, this review highlights the importance of distinguishing constituent-specific mechanisms, exposure plausibility, and attribution directness when evaluating the translational potential of *A. sinensis*.

## 1. Introduction

*Angelica sinensis* (Oliv.) Diels (*A. sinensis*), commonly known as Danggui, is a widely used medicinal herb in traditional Chinese medicine. It has historically been used for blood-nourishing, menstrual disorders, pain, anemia-related conditions, and inflammatory diseases [1,2,3]. Modern studies have reported anti-inflammatory, antioxidant, immunomodulatory, hematopoietic, neuroprotective, vasoprotective, metabolic regulatory, and tissue-repair effects [4,5,6]. These activities have been mainly associated with phthalides, phenolic acids, polysaccharides, volatile oils, and other minor constituents [3,7,8]. Among them, ligustilide (LIG, isomer not specified) and related phthalides, ferulic acid (FA) and other phenolic acids, and *A. sinensis* polysaccharides have received particular attention because of their relevance to inflammation, oxidative stress, vascular function, immune regulation, and cellular homeostasis [9,10,11,12]. Despite extensive pharmacological research, the clinical translation of *A. sinensis* remains limited by several challenges. First, many studies use crude extracts, fractions, polysaccharide preparations, commercial products, or multi-herb formulas rather than chemically defined constituents [2,13,14]. This makes it difficult to attribute observed effects to specific compounds or constituent classes. Such distinction is important because phthalides, phenolic acids, and polysaccharides differ markedly in chemical stability, extraction behavior, pharmacokinetics, tissue distribution, and biological targets [8,10,15,16]. Therefore, evidence obtained from one material type cannot be directly extrapolated to another without considering chemical composition, exposure, and preparation-specific properties.

Second, multiple signaling pathways have been implicated in the pharmacological effects of *A. sinensis*. These include nuclear factor kappa B (NF-κB), mitogen-activated protein kinase (MAPK), phosphoinositide 3-kinase/protein kinase B (PI3K/Akt), Janus kinase/signal transducer and activator of transcription (JAK/STAT), nuclear factor erythroid 2-related factor 2/heme oxygenase 1 (Nrf2/HO-1), apoptosis, autophagy, and ferroptosis-related pathways [4,17,18,19,20,21,22,23]. However, the strength of mechanistic evidence varies considerably. Many studies rely mainly on pathway-marker changes in single cellular or animal models. Few studies link these mechanisms to pharmacokinetic exposure, active metabolites, tissue distribution, or clinically meaningful biomarkers. This gap is particularly relevant for compounds with limited oral bioavailability, rapid metabolism, or chemical instability, such as LIG and FA. Thus, some reported mechanisms may be biologically plausible but not yet pharmacologically exposure-relevant. Clinical interpretation is also complicated by the limited availability of direct single-herb evidence. Although Danggui-containing preparations have been evaluated in gynecological, hematological, cardiovascular, neurological, inflammatory, and supportive-care settings, most clinical studies involve multi-herb formulas rather than *A. sinensis* alone [24,25]. Consequently, the independent contribution of *A. sinensis* remains difficult to determine. Existing clinical evidence is further limited by small sample sizes, heterogeneous formulations, variable quality control, inconsistent endpoints, and insufficient safety reporting [26]. Phthalide-related clinical evidence also requires careful interpretation. For example, butylphthalide demonstrates the drug-development potential of the phthalide scaffold, but its clinical effects should not be directly extrapolated to whole *A. sinensis*, crude extracts, or traditional preparations [27]. Previous reviews have summarized the phytochemistry, pharmacological activities, disease-specific applications, or individual constituent classes of *A. sinensis* [1,2,15,28]. However, several translational issues remain insufficiently addressed. These include material attribution across phthalides, phenolic acids, polysaccharides, whole extracts, and multi-herb formulas; the distinction between causal mechanistic validation and pathway-marker association; the exposure plausibility of in vitro findings; and the extent to which clinical signals can be directly attributed to *A. sinensis*. Therefore, this review reappraises *A. sinensis* through a constituent-specific and exposure-relevant translational framework. It focuses on material attribution, dose–exposure plausibility, direct versus formula-level clinical evidence, and the distinction between standardized herbal preparation development and phthalide-inspired drug discovery.

## 2. Literature Search Strategy and Qualitative Evidence Appraisal

This narrative translational review synthesized evidence on the phytochemistry, pharmacology, pharmacokinetics, safety, clinical evidence, and translational development of *Angelica sinensis*. A narrative design was adopted because the literature is highly heterogeneous, spanning phytochemical, pharmacokinetic, preclinical, formulation, safety, clinical, systematic-review, and multi-herb formula studies that differ in material type, constituent attribution, preparation quality, dose range, exposure information, model system, endpoint selection, and clinical directness. Therefore, quantitative synthesis or meta-analysis was not suitable, and the review focused on qualitative appraisal of material definition, constituent attribution, mechanistic validation, exposure plausibility, and clinical relevance.

PubMed, Web of Science Core Collection, Scopus, ScienceDirect, Google Scholar, and China National Knowledge Infrastructure were searched from database inception to 31 May 2026. Search terms combined botanical and common names, including “*Angelica sinensis*”, “Danggui”, “dong quai”, “Angelicae Sinensis Radix”, and “Radix Angelicae Sinensis”, with constituent- and topic-related terms, including “ligustilide”, “Z-ligustilide”, “phthalide”, “ferulic acid”, “phenolic acid”, “polysaccharide”, “pharmacology”, “mechanism”, “pharmacokinetics”, “bioavailability”, “toxicity”, “clinical trial”, “systematic review”, and “translational pharmacology”.

The search initially yielded 4005 records. After removal of 952 duplicates and preliminary exclusion of clearly irrelevant records, 1872 records underwent title and abstract screening. Records were excluded if they were correction or retraction notices, studies on unrelated species or prescriptions, agricultural or pathogen-control studies without pharmacological relevance, or reports lacking direct relevance to *A. sinensis*, its major constituent classes, or the review topics. Predefined supplementary searches and backward or forward citation tracking were also conducted when needed to support specific translational arguments, including constituent pharmacokinetics, metabolism, tissue distribution, formulation behavior, exposure-based interpretation, phthalide-inspired drug development, safety, herb–drug interactions, evidence-appraisal methods, synergy assessment, network pharmacology, and herbal-intervention reporting.

Overall, 121 references were included: 73 from the primary screening set and 48 from supplementary searches or citation tracking. Eligible studies addressed chemical composition, constituent-specific pharmacology, pharmacokinetics or tissue distribution, dose–exposure relationships, mechanistic validation, safety, clinical application, or translational development. Greater interpretive weight was given to studies with defined materials, identifiable constituents or fractions, exposure-related data, in vivo validation, causal mechanistic testing, target validation, formulation-specific information, or clinically relevant outcomes; studies with poorly defined preparations, insufficient methodological detail, limited translational relevance, or unclear material attribution were used only as contextual evidence.

Given the narrative and translational scope, no Preferred Reporting Items for Systematic Reviews and Meta-Analyses flow diagram, meta-analysis, or comprehensive risk-of-bias assessment was performed. Evidence was appraised using predefined qualitative domains, including material definition, constituent attribution, model relevance, mechanistic support, exposure plausibility, formulation specificity, safety relevance, and clinical directness. Pharmacological studies in Table 1 were evaluated with separate consideration of mechanistic support and exposure plausibility, whereas clinical evidence in Table 2 was interpreted according to attribution directness, formulation type, methodological limitations, endpoint relevance, and clinical translatability.

## 3. Phytochemical Basis of Constituent-Specific Pharmacology

*A. sinensis* contains diverse constituents, including phthalides, phenolic acids, phenylpropanoids, polysaccharides, terpenoids, polyynes, flavonoids, fatty acids, and nitrogen-containing compounds. This chemical complexity supports its broad pharmacological profile but also complicates constituent attribution, quality control, exposure assessment, and translational interpretation. Current evidence identifies phthalides, phenolic acids, particularly FA, and polysaccharides as the principal bioactive classes most consistently associated with its major pharmacological effects. Other constituents may contribute to chemical diversity or complementary activity, but their specific roles remain less defined. Advances in high-performance liquid chromatography (HPLC), ultra-performance liquid chromatography (UPLC), gas chromatography–mass spectrometry (GC–MS), liquid chromatography–mass spectrometry (LC–MS), nuclear magnetic resonance (NMR), and structure–activity analyses have improved characterization of the chemical structures, stability, and bioactivity-related properties of *A. sinensis* constituents. This section summarizes the major and minor constituent classes as the phytochemical basis for constituent-specific pharmacology.

### 3.1. Major Active Constituents

#### 3.1.1. Phthalides

Phthalides are characteristic lipophilic constituents of *A. sinensis* and major components of its essential oil. They share a phthalide, or isobenzofuranone, core but differ in substituent patterns, double bond configuration, and degree of dimerization or oligomerization, thereby contributing to chemical diversity and preparation-dependent variability [29]. Among monomeric phthalides, Z-ligustilide (Z-LIG) is generally regarded as the most abundant and extensively studied compound in *A. sinensis* and related Apiaceae medicinal plants [9]. As a Z-configured butylidene phthalide, Z-LIG contains an unsaturated lactone and conjugated double bonds, which may contribute to its bioactivity but also confer high chemical reactivity and instability [9]. Z-LIG is sensitive to light, heat, oxygen, pH, and solvent conditions and may undergo isomerization, oxidation, hydrolysis, cycloaddition, and polymerization, leading to degradation products and altered phthalide profiles [9,16]. In addition to Z-LIG, *A. sinensis* contains related monomeric phthalide isomers, dimers, trimers, and glycosylated or structurally modified derivatives [30,31,32,33]. Some dimeric and trimeric phthalides may originate from LIG-type precursors through cycloaddition or related transformation processes, linking phthalide instability to structural diversification [30,32]. These compounds may differ in lipophilicity, stability, metabolism, permeability, tissue distribution, and biological activity, whereas their structure–activity relationships, in vivo relevance, and contribution to whole-herb pharmacology remain incompletely defined. Thus, phthalides should be considered not only bioactive and quality-control markers but also chemically dynamic constituents that affect quality control, exposure interpretation, and translational reproducibility.

#### 3.1.2. Ferulic Acid and Related Phenolic Acids

Ferulic Acid (FA) and related phenolic acids are important hydrophilic constituents of *A. sinensis* and contribute to its antioxidant and redox-regulatory potential. These compounds are mainly derived from the phenylpropanoid pathway and typically contain phenolic hydroxyl and methoxy groups within conjugated structures, supporting free radical scavenging and redox modulation [34]. FA is the most representative phenolic acid in *A. sinensis* and is widely used as a marker compound in quality evaluation and chemical studies. Its structural features are associated with antioxidant, anti-inflammatory, and vasoprotective activities [35,36,37]. However, FA is not unique to *A. sinensis*, and its contribution to whole-herb preparations should be interpreted in relation to content, extraction efficiency, metabolism, bioavailability, and matrix effects. Other phenolic acids, including cinnamic acid and vanillic acid, have also been identified, but they are generally less abundant and less extensively studied, and their contribution to the overall pharmacological profile remains unclear [38].

#### 3.1.3. Polysaccharides

*A. sinensis* polysaccharides are high-molecular-weight bioactive fractions most consistently associated with hematopoietic, immunomodulatory, metabolic, and tissue-protective effects [7,15,39]. Rather than single defined compounds, they should be regarded as structurally heterogeneous fractions, because source material and preparation procedures markedly affect molecular weight distribution, monosaccharide composition, uronic acid content, glycosidic linkages, branching degree, and higher-order conformation [4]. Reported polysaccharides are mainly heteropolysaccharides, including acidic forms, and commonly contain glucose, galactose, arabinose, rhamnose, xylose, mannose, fucose, and uronic acids such as galacturonic and glucuronic acids, with substantial variation in molar ratios [39,40,41]. This heterogeneity limits cross-study comparison and complicates quality control, dose interpretation, and structure–activity analysis. Biological activity appears to be influenced by molecular weight, uronic acid content, linkage type, branching pattern, and conformation [4,41]. Extraction, deproteinization, purification, fractionation, and drying may further alter both structure and activity [7,40,42]. Therefore, evaluation of *A. sinensis* polysaccharides should integrate source, preparation, structural characterization, and functional testing, and standardized polysaccharide profiling is essential for clarifying their pharmacological relevance and translational potential.

### 3.2. Other Reported Phytochemical Constituents

In addition to phthalides, phenolic acids, and polysaccharides, *A. sinensis* contains other minor constituents, including volatile oil components, terpenoid-related compounds, polyynes, flavonoid-related metabolites, fatty acids and their esters, sterol-like compounds, and nitrogen-containing small molecules [1,43,44,45]. Metabolomic and GC–MS-based studies indicate that these minor constituents vary with source, cultivation status, medicinal part, and developmental stage, further supporting the material-dependent chemical complexity of *A. sinensis* [43,44]. Although these constituents may contribute to aroma, matrix effects, or complementary activity, most remain insufficiently characterized and currently lack enough evidence to be considered major bioactive determinants [1,3].

## 4. Pharmacokinetic and In Vivo Distribution Characteristics of Major Active Components

The major bioactive constituent classes of *A. sinensis* differ markedly in chemical stability, absorption, metabolism, tissue distribution, and systemic exposure. These differences are critical for interpreting constituent-specific pharmacology, because in vitro effects cannot be directly extrapolated to in vivo efficacy unless tested concentrations are compatible with achievable exposure. Pharmacokinetic and distributional evidence therefore provides an essential framework for evaluating whether reported mechanisms are pharmacologically plausible, exposure-relevant, or mainly mechanistic amplification models.

### 4.1. Phthalides: Instability, Low Oral Exposure, and Formulation-Dependent Distribution

Phthalide derivatives are lipophilic small molecules that may be rapidly absorbed and widely distributed, but their systemic exposure is limited by chemical instability, rapid metabolism, first-pass elimination, and preparation-dependent matrix effects [46]. Z-LIG is the most extensively studied phthalide relevant to *A. sinensis.* Because Z-LIG also occurs in other phthalide-rich Apiaceae medicinal plants, several pharmacokinetic and brain-delivery studies have used Rhizoma Chuanxiong or isolated LIG rather than *A. sinensis* preparations. Tissue-distribution studies detected LIG in rat brain after oral administration, with relatively higher levels in the cerebellum and cerebrum than in several peripheral tissues, suggesting that LIG-type phthalides may access the central nervous system [47]. Intranasal administration further produced rapid brain detection and measurable extracellular unbound LIG in freely moving rats, indicating that the administration route can substantially alter central exposure [48,49]. In experimental cerebral ischemia, intranasal Z-LIG showed neuroprotective effects involving nuclear factor erythroid 2-related factor 2 (Nrf2)- and heat shock protein 70 (HSP70)-related mechanisms, supporting route-specific central pharmacology [50]. Low oral exposure remains a major constraint for interpreting Z-LIG-related pharmacology. In the rat pharmacokinetic study by Yan et al., LIG was structurally defined as Z-3-butylidene-4,5-dihydroisobenzofuranone and is therefore discussed here as Z-LIG or LIG-containing preparations in that experimental context [10]. Its oral bioavailability was reported as 2.6% relative to intravenous administration. Intravenous LIG was formulated in Pharmatek formulation-6, whereas oral LIG was administered in normal saline containing 3% Tween 80 at 100, 360, or 500 mg/kg. Reliable oral pharmacokinetic parameters were mainly obtained at 500 mg/kg; at 100 mg/kg, plasma concentrations were below the detectable limit. At 500 mg/kg, the maximum plasma concentration (Cmax) was approximately 0.66 μg/mL, equivalent to about 3.5 μM, with a time to maximum plasma concentration (Tmax) of approximately 0.36 h [10]. Thus, the 2.6% bioavailability should be interpreted as an intravenous-referenced, formulation-specific estimate in rats rather than a general benchmark for all *A. sinensis* preparations.

These findings indicate that pharmacokinetic data from purified Z-LIG or LIG-containing preparations, volatile oils, crude extracts, and multi-herb preparations should not be treated as interchangeable. Route-specific delivery, such as intranasal administration, may partly overcome systemic exposure constraints, but such findings should not be directly extrapolated to conventional oral *A. sinensis* preparations. High-concentration in vitro studies, particularly those using ≥50 μM Z-LIG, substantially exceed reported oral systemic exposure and should be regarded mainly as mechanistic amplification models unless supported by tissue exposure, metabolite activity, formulation-enhanced bioavailability, route-specific delivery, or pharmacokinetic/pharmacodynamic (PK/PD) evidence. Future studies should distinguish parent-compound effects from metabolite-mediated effects and determine whether improved stability or delivery systems can produce reproducible pharmacodynamic responses under exposure-relevant conditions.

### 4.2. Ferulic Acid and Phenolic Acids: Rapid Absorption, Extensive Conjugation, and Attribution Caveats

Phenolic acids, represented by ferulic acid (FA), are generally more hydrophilic than phthalides and can be absorbed rapidly after gastrointestinal administration. However, FA is not specific to *Angelica sinensis* and is widely present in foods, cereals, and other medicinal or dietary plants. Therefore, pharmacokinetic and mechanistic findings from purified FA, dietary sources, or non-*A. sinensis* materials should be interpreted as FA-associated evidence rather than direct evidence for *A. sinensis*-derived FA unless the tested material, source, and exposure are clearly defined.

After absorption, FA is extensively subject to phase II metabolism, especially glucuronidation and sulfation, resulting in relatively low systemic exposure to the free parent compound [35,51,52,53]. Consistent with this metabolic pattern, FA sulfate has also been detected in human serum after oral intake of a willow bark extract [54], further illustrating that much of the available FA exposure evidence comes from broader phenolic acid contexts rather than from *A. sinensis* preparations specifically. This distinction is important because many cellular studies evaluate unconjugated FA at concentrations that may exceed physiologically relevant free plasma exposure.

In a rat in situ gastric administration study, 2.25 μmol FA, corresponding to approximately 8 μmol/kg body weight, was administered in 0.5 mL physiological saline and retained in the stomach for 25 min. Under these conditions, total FA in celiac arterial plasma reached approximately 9.2 μM, whereas free FA accounted for only about 6.2% of total exposure, corresponding to approximately 0.6 μM [51,52]. These data indicate that FA can be rapidly absorbed, but circulating FA is present predominantly as conjugated metabolites rather than as the free parent compound. Accordingly, in vitro effects observed only at high micromolar concentrations of unconjugated FA, such as 25–200 μM or higher, should be interpreted cautiously as potentially supraphysiological for systemic free FA exposure.

This limitation does not exclude pharmacological relevance, but it narrows the interpretation. Conjugated metabolites may contribute directly or after local deconjugation; intestinal luminal or mucosal exposure may exceed systemic free plasma levels, making barrier-protective and gut-associated effects more plausible; and herbal matrices or formulations may modify absorption, metabolism, and tissue distribution. However, when FA studies are cited to support *A. sinensis* pharmacology, attribution should be made cautiously and should consider FA content in the preparation, extraction efficiency, matrix effects, source material, free versus conjugated exposure, and tissue or local concentration. Future studies should quantify both free and conjugated FA in A. sinensis-derived preparations, evaluate metabolite activity, and align in vitro concentrations with measured plasma, tissue, intestinal, or local exposure.

### 4.3. Polysaccharides: Non-Classical Absorption and Preparation-Dependent Behavior

In contrast to phthalides and phenolic acids, *A. sinensis* polysaccharides are structurally heterogeneous macromolecular fractions whose absorption, distribution, and systemic mechanisms remain less clearly defined. Current evidence is mainly derived from labeled-polysaccharide studies, which suggest that absorption may involve endocytosis, transepithelial transport, or gut-associated immune pathways [55,56]. However, conventional small-molecule pharmacokinetic concepts are not fully applicable to polysaccharides because their biological effects may depend on molecular weight, branching degree, uronic acid content, conformation, gut retention, microbiota interactions, and immune recognition. The preparation-dependent nature of polysaccharides further complicates pharmacokinetic interpretation. Extraction, purification, deproteinization, fractionation, and drying can generate fractions with distinct molecular weight distributions and structural features, thereby influencing absorption, biodistribution, immunomodulatory activity, hematopoietic effects, metabolic regulation, and microbiota-related responses. Human pharmacokinetic data for *A. sinensis* polysaccharides remain limited, and direct links between specific structural fractions, systemic exposure, and pharmacodynamic outcomes are still lacking. Polysaccharide-related pharmacology should therefore be interpreted through a broader exposure framework that includes intestinal retention, epithelial transport, gut immune activation, microbiota modulation, and systemic immune or metabolic responses. Future studies should use structurally traceable or labeled polysaccharide fractions, combined with functional assays, to clarify whether observed effects are mediated by absorbed polysaccharide fragments, gut-associated signaling, microbiota-derived metabolites, or indirect host responses.

### 4.4. Implications for Interpreting In Vitro Pharmacology

The pharmacokinetic profiles summarized above provide a framework for interpreting in vitro pharmacological findings on *A. sinensis*. Phthalides and phenolic acids may be absorbed after administration, but their translational relevance is constrained by instability, first-pass metabolism, conjugation, and low free parent-compound exposure. In contrast, polysaccharides cannot be evaluated using the same plasma concentration-based framework, because their effects may involve gut retention, epithelial interaction, microbiota modulation, immune recognition, or preparation-dependent macromolecular behavior.

**Figure 1:**
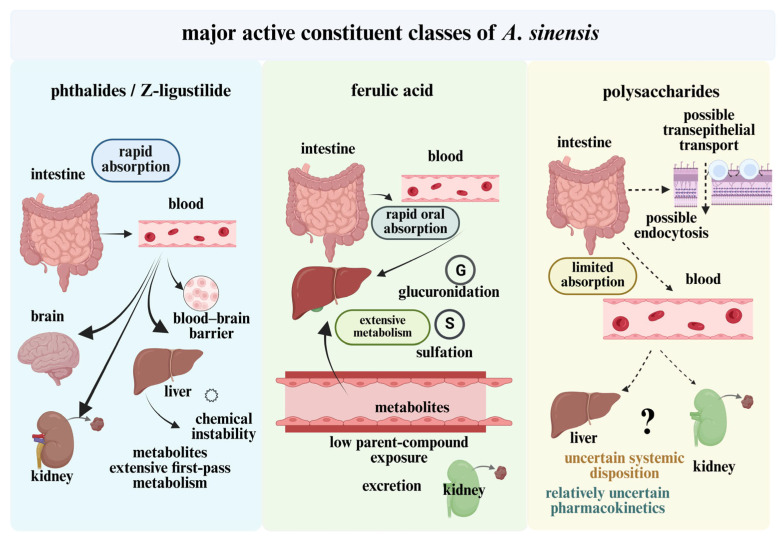
Proposed schematic summary of tissue distribution and exposure-related constraints of the major bioactive constituent classes of *A. sinensis* in vivo. This framework is based on available pharmacokinetic and tissue-distribution evidence and is intended as an interpretive guide rather than definitive tissue-target attribution. (Created in BioRender. jialian, S. (2026) https://BioRender.com/s04ytoo) access on 9 July 2026.

Accordingly, in vitro concentrations should not be interpreted uniformly across constituent classes. For Z-LIG and FA, high-micromolar cellular models may identify possible signaling pathways but do not necessarily represent exposure-relevant pharmacology after conventional oral administration. For polysaccharides and complex preparations, the key limitations are often insufficient structural definition, uncertain local or systemic disposition, and poor preparation comparability. Therefore, pharmacological findings should be interpreted in relation to exposure route, parent or metabolite contribution, tissue or local concentration, formulation effects, and preparation identity. The putative in vivo distribution and exposure-related constraints of the major constituent classes are summarized in Figure 1.

## 5. Pharmacological Properties of *Angelica sinensis*

Beyond its traditional uses in tonifying blood, regulating menstruation, and relieving pain, *A. sinensis* has been investigated in cellular and animal models of neurological injury, cardiovascular injury, intestinal inflammation, metabolic dysfunction, tissue repair, hematopoietic impairment, and tumor-related conditions [11,28]. Reported activities include anti-inflammatory, antioxidant, immunomodulatory, neuroprotective, metabolic regulatory, hematopoietic-supportive, tissue-protective, and antitumor-related effects. However, the translational relevance of these activities varies substantially because the major constituent classes differ in material definition, chemical stability, metabolism, systemic exposure, tissue distribution, and preparation dependence.

To clarify the preclinical evidence without over-interpreting heterogeneous studies, Table 1 provides a condensed summary appraisal by pharmacological category and component/preparation class, whereas full study-level details are provided in Supplementary Table S1. The appraisal considered constituent attribution, model relevance, mechanistic support, exposure plausibility, and clinical translatability. Mechanistic support was evaluated separately from exposure plausibility because causal pathway validation does not necessarily indicate that the tested concentration is achievable in vivo or relevant to conventional *A. sinensis* exposure. Therefore, studies with strong mechanistic validation but exposure-limited concentrations were interpreted as mechanistically informative rather than directly translational.

Across the 41 representative study-level entries summarized in Supplementary Table S1, only a small subset was judged directly exposure-plausible at the tested cellular concentration. Several additional studies were considered marginal, exposure-limited, dose-based but pharmacokinetically uncertain, or route-, formulation-, local-delivery-, or combination-specific. Many entries were not directly assessable by classical plasma concentration-based comparison, particularly those involving polysaccharides, crude extracts, oils, complex preparations, or insufficiently characterized materials. These categories represent qualitative translational judgments rather than formal risk-of-bias ratings or objective scoring outcomes.

**Figure 2:**
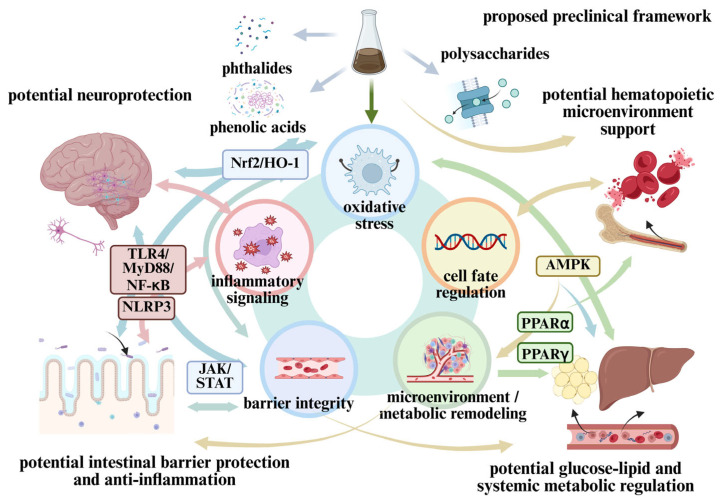
Proposed mechanistic framework of *A. sinensis* bioactive constituents, highlighting major regulatory pathways and disease-related contexts reported in preclinical studies. The indicated links are schematic hypotheses derived from heterogeneous experimental models and require exposure-matched, preparation-specific, and clinical validation. (Created in BioRender. jialian, S. (2026) https://BioRender.com/s04ytoo) access on 9 July 2026.

This appraisal indicates that many reported mechanisms remain valuable for pathway discovery, but only a minority are currently supported by exposure-compatible or preparation-specific evidence. Translational interpretation therefore requires additional support from tissue exposure, metabolite activity, formulation-specific pharmacokinetics, local concentration measurements, structurally defined preparations, or integrated pharmacokinetic/pharmacodynamic validation. The major regulatory pathways and disease-related contexts are summarized as a proposed mechanistic framework in Figure 2, and the constituent-specific appraisal is summarized in Table 1.

**Table 1:** Summary translational appraisal of pharmacological studies on *Angelica sinensis* constituents and preparations.

Category	Component/Preparation Class	Studies	Model and Representative Dose/Concentration Range	Mechanistic Support	Exposure Plausibility	Translational Interpretation and Main Effect
Neuroprotection	*A. sinensis* polysaccharide(s) (ASP)	[57]	Amyloid beta 25–35-induced Alzheimer’s disease rats; 50 mg/kg	Strong; in vivo pathway inhibition supports causality	Not assessable using classical small-molecule pharmacokinetics; in vivo dosing supports biological relevance	Moderate–strong, preparation-specific; brain-derived neurotrophic factor/tropomyosin receptor kinase B/cyclic adenosine monophosphate response element-binding protein signaling
Neuroprotection	Phthalides: Z-ligustilide (Z-LIG) and ligustilide with unspecified isomer	[18,58,59,60]	Oxidative, ischemic, spinal cord injury, and intervertebral disc degeneration models; 0.1–5 μg/mL, 1–50 μM, and 10–50 mg/kg in selected in vivo models	Moderate to strong across grouped studies; several studies used pathway inhibitors, gene silencing, or autophagy/mitophagy validation	Low-micromolar models are more exposure-compatible; 10–50 μM models are generally exposure-limited without tissue pharmacokinetic support	Mechanistically informative but exposure-constrained; mitochondrial protection, anti-apoptosis, autophagy/mitophagy regulation, and anti-pyroptosis
Neuroprotection	*A. sinensis* extract	[61,62]	Cerebral ischemia and neurogenesis models; 0.25–1 g/kg	Strong at the extract level, including pathway blockade or pathway-level validation	Not assessable; crude extract composition and constituent exposure are preparation-dependent	Moderate to moderate–strong, formulation-specific; p38 mitogen-activated protein kinase-related anti-apoptosis and cyclic adenosine monophosphate response element-binding protein/brain-derived neurotrophic factor-related neurogenesis
Neuroprotection	Ferulic acid (FA)	[19]	Chronic constriction injury rats with Schwann cell and microglia models; 100 mg/kg and 2 μM	Moderate–strong; ribonucleic acid sequencing and inhibitor-supported validation	Marginal; 2 μM exceeds reported free FA exposure but is closer than high-micromolar models	Moderate–strong as FA-associated evidence, but not directly *A. sinensis*-specific unless source attribution, preparation matrix, and free/conjugated FA exposure are defined; toll-like receptor 4/myeloid differentiation primary response 88/nuclear factor kappa B inhibition
Metabolic regulation	ASP	[63,64]	KKAy diabetic mice, high-fat diet-fed mice, and insulin-resistant HepG2 cells; 80–400 mg/kg/day and 50–200 μg/mL	Moderate–low to moderate–strong across grouped studies; evidence ranges from gut microbiota/metabolomics association to pathway-supported insulin-signaling validation	Not assessable using classical small-molecule pharmacokinetics; gut-associated exposure may be relevant, whereas in vitro ASP concentrations lack direct exposure linkage	Moderate but preparation-dependent; gut microbiota remodeling, adiponectin/sirtuin 1/adenosine monophosphate-activated protein kinase regulation, peroxisome proliferator-activated receptor gamma modulation, phosphoinositide 3-kinase/protein kinase B-related insulin signaling, and improved glucose/lipid metabolism
Metabolic regulation	FA	[65,66,67]	High-fat diet-induced fatty liver, endurance model, HepG2 cells, and C2C12 myotubes; 0.5% diet, 0.5–200 μM, and 100 mg/kg/day	Moderate–low to moderate–strong across grouped studies	0.5 μM is exposure-compatible; 50–200 μM is likely supraphysiological for free FA	Mixed translational relevance and primarily FA-associated rather than *A. sinensis*-specific evidence; β-oxidation, mitochondrial homeostasis, adenosine monophosphate-activated protein kinase/peroxisome proliferator-activated receptor gamma coactivator 1-alpha/nuclear factor erythroid 2-related factor 2 signaling
Intestinal inflammation and barrier injury	FA	[20,68,69]	Radiation injury, Caco-2 barrier dysfunction, and arsenite-induced colon injury; 10–100 mg/kg and 25–100 μM	Moderate to moderate–strong across grouped studies	Systemic free FA exposure is often limited, but intestinal luminal or mucosal exposure may be more relevant	Moderate but requires local tissue exposure validation; these findings should be interpreted as FA-associated barrier-protective evidence rather than direct *A. sinensis*-specific evidence unless source/preparation attribution is defined; tight junction protection, phosphoinositide 3-kinase/protein kinase B-related barrier restoration, and anti-inflammatory/antioxidant effects
Intestinal inflammation and barrier injury	ASP-based, plant-part-specific, or modified polysaccharide preparations	[70,71,72]	Dextran sulfate sodium colitis, IPEC-J2 cells, and alcoholic fatty liver disease-associated gastrointestinal injury; approximately 10–150 mg/kg and 5–20 μg/mL	Moderate to moderate–strong across grouped studies	Preparation-, plant-part-, or formulation-specific; findings should not be generalized across ASP preparations without structural and formulation comparability	Moderate, preparation-specific; toll-like receptor 4/myeloid differentiation primary response 88/nuclear factor kappa B inhibition, short-chain fatty acid-related barrier homeostasis, and microbiota modulation
Intestinal inflammation and barrier injury	Angelica oil	[73]	Dextran sulfate sodium-induced colitis; 10–40 mg/kg	Moderate–low; mainly omics-associated evidence	Not assessable; oil composition and constituent exposure are variable	Moderate–low, formulation-dependent; microbiota and sphingolipid metabolism regulation
Hematopoietic microenvironment regulation	ASP and Angelica polysaccharide iron complex	[74,75,76,77,78]	Cell/co-culture models and anemia or hematopoietic-aging animal models; 100 μg/mL, 200 mg/kg–1 g/kg, and 12.5–50 mg Fe/kg for the iron complex	Low to moderate across grouped studies; strongest where pathway support is available	Classical small-molecule pharmacokinetic comparison is not applicable; iron complex should be treated as a distinct formulation	Low–moderate to moderate; hematopoietic niche preservation, delayed hematopoietic stem and progenitor cell senescence, hepcidin/ferroportin 1 regulation, and hypoxia-inducible factor 2 alpha/erythropoietin-related erythropoietic support
Other tissue-protective effects	ASP	[21,79,80,81,82,83]	Cardiac, liver, fibrosis, osteoarthritis, inflammatory cell, and glioma models; 1–200 μg/mL and 50–400 mg/kg/day	Moderate–low to strong across grouped studies	Not assessable using classical pharmacokinetics; in vitro ASP concentrations lack direct exposure linkage	Preparation-specific; activating transcription factor 6/adenosine monophosphate-activated protein kinase cardioprotection, extracellular signal-regulated kinase 1/2-autophagy regulation, nuclear factor erythroid 2-related factor 2 activation, interleukin-22/signal transducer and activator of transcription 3 anti-fibrotic effects, and preliminary anti-inflammatory or antitumor signals
Other tissue-protective effects	FA	[84,85,86,87,88]	Tumor, nephropathy, pneumonia, liver injury, and acute lung injury models; 0.1–800 μM and 20–200 mg/kg/day	Moderate–low to strong across grouped studies	High-micromolar FA models are exposure-limited; the 0.1 μM acute lung injury cell model is more exposure-compatible, although tissue pharmacokinetic/pharmacodynamic validation is still needed	Mixed translational relevance and primarily FA-associated rather than *A. sinensis*-specific evidence; mitogen-activated protein kinase/autophagy-associated apoptosis, inflammasome regulation, peroxiredoxin 1-toll-like receptor 4-nuclear factor kappa B inhibition, and anti-ferroptosis
Other disease-specific or route-specific effects	Phthalides: N-butylidenephthalide, Z-LIG, and ligustilide with unspecified isomer	[22,89,90,91]	Vascular inflammation, breast cancer, ultraviolet B injury, and osteoarthritis models; 1–100 μM or μg/mL, and intra-articular 75–150 μM	Moderate to strong across grouped studies	Route-specific or compound-specific; topical or intra-articular exposure should not be extrapolated to oral *A. sinensis* exposure	Low–moderate to moderate; nuclear factor erythroid 2-related factor 2/heme oxygenase 1 activation, caspase-dependent apoptosis, nuclear factor kappa B inhibition, and c-Jun N-terminal kinase/p38 mitogen-activated protein kinase inhibition
Combination-specific antitumor effect	ASP plus cisplatin	[23]	Cisplatin-resistant ovarian cancer model; ASP 200 μg/mL and in vivo combination dosing as reported	Strong; glutathione peroxidase 4 rescue and ferrostatin-1 support ferroptosis mechanism	Combination-specific; ASP exposure and dose–response linkage remain unclear	Moderate but combination-specific; glutathione peroxidase 4-dependent ferroptosis and enhanced cisplatin sensitivity

Note: Table 1 summarizes the study-level appraisal provided in Supplementary Table S1. Mechanistic support and exposure plausibility were appraised separately to avoid equating causal pathway validation with translational relevance. Mechanistic support was classified qualitatively as strong, moderate–strong, moderate, moderate–low, or low according to the extent of in vivo confirmation, causal testing, pathway validation, and model relevance. Exposure plausibility was classified as plausible, marginal, limited, or not assessable according to whether the tested concentration or dose was compatible with reported systemic, tissue, intestinal, local, or formulation-related exposure. For polysaccharides, exposure plausibility was interpreted according to preparation identity, gut-associated exposure, immune recognition, microbiota-related effects, and in vivo functional dosing rather than classical plasma pharmacokinetics. For ferulic acid-related entries, the evidence was interpreted as ferulic acid-associated rather than A. sinensis-specific unless source, matrix, and exposure attribution were defined. These categories represent qualitative translational judgments made by the writing team using predefined appraisal domains. No independent duplicate assessment or inter-rater reliability analysis was performed; therefore, the categories should not be interpreted as formal risk-of-bias ratings, GRADE ratings, or objective scoring outcomes.

### 5.1. Regulation of Cell Fate, Inflammation, and Redox Homeostasis

Dysregulated cell fate, persistent inflammation, oxidative stress, and mitochondrial dysfunction are recurrent pathological processes investigated in pharmacological studies of *A. sinensis* constituents and preparations. Reported pathways include apoptosis, autophagy/mitophagy, ferroptosis, pyroptosis, nuclear factor kappa B (NF-κB), mitogen-activated protein kinase (MAPK), NOD-like receptor family pyrin domain-containing 3 (NLRP3) inflammasome, Janus kinase/signal transducer and activator of transcription (JAK/STAT), and nuclear factor erythroid 2-related factor 2/heme oxygenase 1 (Nrf2/HO-1) signaling. However, these pathway links should be interpreted as preclinical and model-dependent rather than established therapeutic mechanisms, because the strength of evidence varies according to material type, tested concentration, exposure route, and validation strategy.

In non-neoplastic injury models, ligustilide-type phthalides have been reported to reduce apoptosis and promote protective autophagy or mitophagy in neural injury and intervertebral disc degeneration models. Mechanistic support includes pharmacological inhibition or gene-silencing approaches involving adenosine monophosphate-activated protein kinase/mammalian target of rapamycin (AMPK/mTOR), B-cell lymphoma 2-interacting protein 3 (BNIP3), and autophagy-related protein 5 (Atg5)-related pathways [18,58,59,60]. These findings are mechanistically informative, but their translational interpretation should remain exposure-aware, because some cellular concentrations exceed reported oral systemic exposure for ligustilide-type phthalides. *A. sinensis* polysaccharides have also been reported to reduce apoptosis and modulate autophagy in osteoarthritis chondrocytes, although the evidence remains preparation-specific and requires further in vivo confirmation [21]. Tumor-related models show a different, context-dependent pattern. N-butylidenephthalide has been associated with apoptosis and radiosensitization in breast cancer cells, whereas *A. sinensis* polysaccharides combined with cisplatin have been linked to glutathione peroxidase 4 (GPX4)-dependent ferroptosis in cisplatin-resistant ovarian cancer models [23,89]. These findings should not be generalized to tissue-protective settings, because the desired direction of cell-fate regulation differs between injury repair and antitumor contexts. Inflammatory and redox regulation have mainly been linked to NF-κB, MAPK, NLRP3 inflammasome, JAK/STAT, and Nrf2/HO-1 signaling. FA-associated studies have reported inhibition of toll-like receptor 4/myeloid differentiation primary response 88/NF-κB signaling in sciatic nerve injury and peroxiredoxin 1–toll-like receptor 4-related regulation in lipopolysaccharide-induced pneumonia or macrophage models [19,86]. These findings support FA-associated anti-inflammatory mechanisms, but they should not be interpreted as *A. sinensis*-specific unless the FA source, preparation matrix, and free or conjugated exposure are defined. Similarly, FA-related NLRP3, JAK/STAT, Nrf2, and ferroptosis-related findings should be interpreted in light of the limited systemic exposure of free FA and the frequent use of purified FA or non-*A. sinensis* experimental contexts [20,84,85,86,87,88]. *A. sinensis* extracts and ligustilide-type phthalides have also been associated with MAPK- and Nrf2/HO-1-related effects, although pathway direction appears model-dependent and route-dependent [22,61,62,81,90,91]. Overall, the available evidence supports plausible roles for *A. sinensis* constituents and FA-associated mechanisms in cell fate and inflammatory-redox regulation. Nevertheless, these mechanisms remain primarily preclinical hypotheses, and their translational relevance should be interpreted together with exposure plausibility, preparation specificity, and the study-level appraisal summarized in Table 1 and Supplementary Table S1.

### 5.2. Regulation of Barrier Function, Metabolism, and Hematopoietic Microenvironment

*A. sinensis* constituents and preparations have also been investigated for effects on epithelial barrier integrity, metabolic homeostasis, gut microbiota regulation, and hematopoietic support. These findings are relevant to intestinal injury, metabolic disorders, anemia-related conditions, and hematopoietic dysfunction, but they should be interpreted as constituent-class- and preparation-specific signals rather than generalized evidence for all *A. sinensis* preparations. FA-associated studies have reported preservation of intestinal barrier function through restoration of tight junction proteins and phosphoinositide 3-kinase/protein kinase B-related signaling [20,68,69]. Such effects may be more plausible in intestinal settings, where luminal or mucosal exposure could exceed systemic free plasma concentrations. However, because FA is not unique to *A. sinensis* and many FA studies use purified FA or non-*A. sinensis* contexts, these findings should be described as FA-associated evidence unless source attribution, preparation matrix, and local exposure are demonstrated. Polysaccharide preparations and related formulations have been linked to mucosal microenvironment regulation, toll-like receptor 4/myeloid differentiation primary response 88/nuclear factor kappa B inhibition, short-chain fatty acid-associated responses, and gut microbiota remodeling [70,71,72]. Angelica oil has also been associated with microbiota and sphingolipid metabolism changes [73]. These findings remain preparation- or formulation-specific, particularly when modified polysaccharides, nanoparticles, plant-part-specific polysaccharides, oils, or whole extracts are used. In metabolic and hematopoietic models, FA has been associated with fatty acid oxidation, mitochondrial function, and energy-regulatory signaling [65,66,67], whereas *A. sinensis* polysaccharides have been linked to insulin sensitivity, gut microbiota modulation, iron mobilization, erythropoietic support, and bone marrow microenvironment regulation [63,64,74,75,76,77,78]. Overall, barrier-, metabolic-, and hematopoietic-related findings should be framed as preclinical translational hypotheses requiring chemically defined materials, exposure-relevant testing, and preparation-specific validation.

### 5.3. Putative Constituent-Class Complementarity and Exposure-Relevant Interpretation

**Figure 3:**
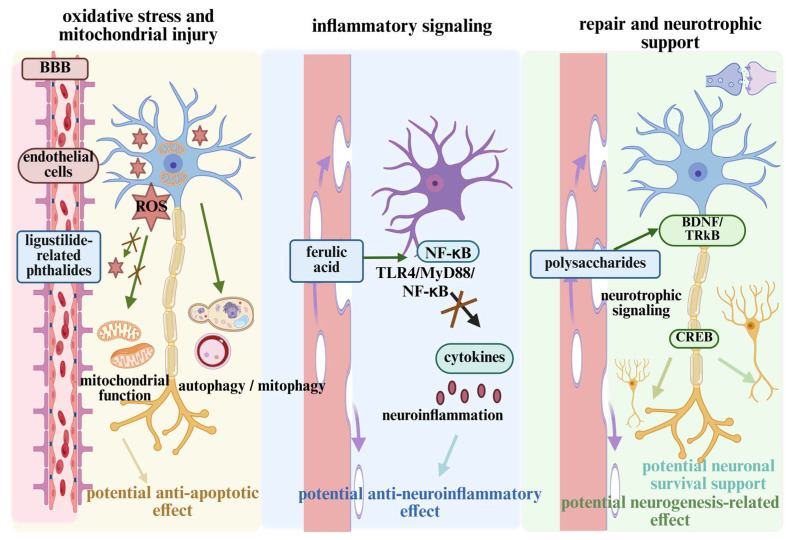
Proposed neuroprotective roles of *A. sinensis* bioactive constituents. The depicted pathways are schematic and hypothesis-generating, remain primarily supported by preclinical models, and should not be interpreted as established mechanisms without pharmacokinetically plausible exposure and in vivo validation. (Created in BioRender. jialian, S. (2026) https://BioRender.com/s04ytoo) access on 9 July 2026.

**Figure 4:**
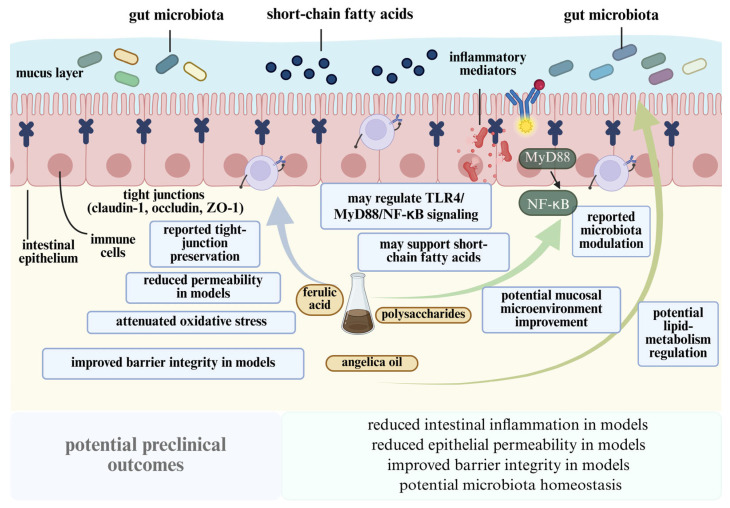
Proposed effects of *A. sinensis* active constituents on intestinal inflammation and epithelial barrier dysfunction. The arrows and pathway links are schematic representations of experimental findings and should not be interpreted as established clinical mechanisms or proven therapeutic effects. (Created in BioRender. jialian, S. (2026) https://BioRender.com/s04ytoo) access on 9 July 2026.

**Figure 5:**
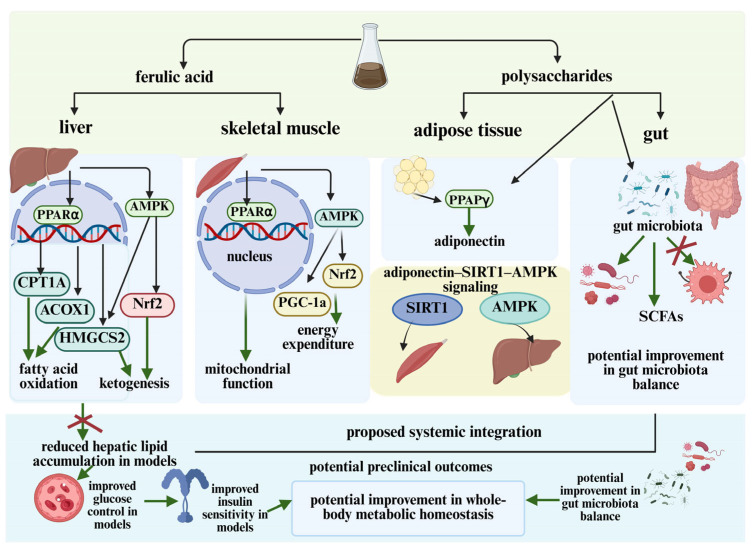
Proposed effects of *A. sinensis* active constituents on glucose and lipid metabolism. The depicted relationships summarize preclinical, hypothesis-generating evidence; direct evidence for coordinated multi-component interaction remains limited and requires chemically defined, exposure-matched validation. (Created in BioRender. jialian, S. (2026) https://BioRender.com/s04ytoo) access on 9 July 2026.

**Figure 6:**
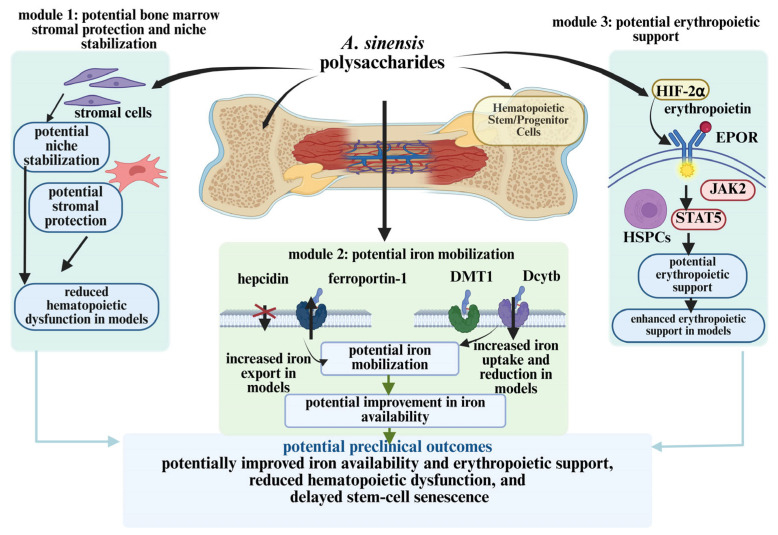
Proposed effects of *A. sinensis* polysaccharides on the hematopoietic microenvironment. The diagram is a schematic interpretation of mainly preclinical evidence and requires confirmation using structurally characterized polysaccharide preparations, exposure-relevant dosing, and clinically meaningful endpoints. (Created in BioRender. jialian, S. (2026) https://BioRender.com/s04ytoo) access on 9 July 2026.

The constituent-specific evidence summarized above suggests a putative division of biological roles among major *A. sinensis* constituent classes. Phthalides, FA-related phenolic acids, and polysaccharides have been associated with overlapping but distinguishable biological processes across experimental models. However, these patterns should be interpreted as hypothesis-generating evidence of potential complementarity, not as proof of coordinated multi-component action or pharmacological synergy. Most available studies examine isolated constituents, extracts, polysaccharide fractions, or formulas in separate models, using different preparations, doses, endpoints, and validation strategies. Whole-extract studies may support preparation-level activity, but they do not resolve the relative contributions of LIG, FA, polysaccharides, and other constituents unless material composition, constituent exposure, and fraction-specific effects are directly characterized. Validation of complementarity requires chemically characterized mixtures, fraction-recombination or component-ablation designs, exposure-matched dosing, and quantitative interaction analyses [92,93,94]. Figure 3, Figure 4, Figure 5 and Figure 6 therefore summarize representative preclinical hypotheses rather than established therapeutic pathways.

## 6. Clinical Evidence and Clinical Applications

Traditional applications of *A. sinensis* in gynecology, pain, blood deficiency-related conditions, and postpartum recovery provide a historical basis for selecting modern research indications, but they do not constitute direct evidence of therapeutic efficacy [1,2,3]. As summarized in Table 2, available human evidence can be broadly divided into direct evidence from single-herb or relatively defined preparations and indirect evidence from multi-herb formulas. Direct evidence allows clearer attribution to *A. sinensis* but remains sparse, whereas formula-level evidence is more abundant but is limited by indirect attribution, heterogeneous interventions, and variable methodological quality. Therefore, clinical findings should be interpreted according to preparation type, study design, endpoint relevance, and attribution directness.

**Table 2:** Clinical applications of *A. sinensis*: representative evidence, methodological limitations, qualitative evidence appraisal, and key conclusions.

Evidence Category	Representative Evidence	Methodological Appraisal	Major Evidence Limitations	Qualitative Confidence in Interpretation	Key Conclusion
Traditional use	Long-standing use in gynecology, blood deficiency-related conditions, pain, and postpartum recovery	Not based on controlled clinical studies; risk-of-bias (RoB) domains not applicable	Indirectness; no direct human efficacy evidence	Not formally appraised	Provides historical clinical context, but not direct evidence of efficacy
Single herb (*A. sinensis*)	Ref. [24]: randomized, double-blind, placebo-controlled trial in postmenopausal women	Randomization and blinding were reported; allocation concealment was unclear; objective outcomes were used	Risk of bias; imprecision; limited sample size	Relatively low (randomized and blinded design, but downgraded for unclear allocation concealment, single small trial, and insufficient power)	No significant benefit over placebo; no clear estrogen-like effect
Defined preparation (Danggui injection)	Ref. [95]: ulcerative colitis study of Danggui injection	Primarily assessed biomarkers; randomization, allocation concealment, and blinding were unclear	Risk of bias; indirectness; imprecision; insufficient clinical endpoint evidence	Very limited (exploratory biomarker-focused evidence with insufficient reporting of core trial-design features)	Exploratory biomarker changes were observed, but clinical efficacy remains unconfirmed
Formula: Danggui Shaoyao San (primary dysmenorrhea)	Ref. [96]: systematic review/meta-analysis of four randomized controlled trials (RCTs)	Included trials were small and generally at high risk of bias; reporting of randomization, concealment, and blinding was poor	Risk of bias; inconsistency; imprecision; indirectness	Very limited (small evidence base with high risk of bias and indirect attribution to *A. sinensis*)	Suggestive benefit for dysmenorrhea, but evidence is insufficient for firm conclusions
Formula: Danggui Buxue Tang (menopausal symptoms)	[97,98,99]	Two randomized double-blind trials support symptom benefit; later follow-up evidence was non-confirmatory	Inconsistency; imprecision; indirectness	Relatively low (randomized double-blind evidence is available, but confidence is downgraded for inconsistent findings and indirect attribution to *A. sinensis*)	May improve some menopausal symptoms, but effects on vasomotor symptoms are limited or inconsistent
Formula: Xiao Yao San disorders of gut–brain interaction (DGBI)/functional gastrointestinal disorders (FGIDs)	Ref. [100]: systematic review/meta-analysis of 48 randomized controlled trials	Large evidence base, but underlying trials were methodologically weak and often poorly reported	Risk of bias; inconsistency; indirectness; potential publication bias	Relatively low (large trial base and meta-analytic evidence, but downgraded for weak trial quality and formula-level indirectness)	Suggests improvement in gastrointestinal and mood-related symptoms, but evidence is not confirmatory
Formula: Xiao Yao San (anxiety)	Ref. [101]: systematic review/meta-analysis of 14 trials	Many included studies had high or moderate risk of bias; most were adjunctive-treatment designs	Risk of bias; indirectness; imprecision	Relatively low (systematic-review evidence is available, but most studies were adjunctive and methodologically limited)	May improve efficacy and safety as adjunct therapy, but certainty remains limited
Formula: Danggui Liuhuang Decoction (hyperthyroidism)	Ref. [25]: systematic review/meta-analysis of randomized controlled trials	Included trials were generally of low methodological quality, with inadequate reporting of key design features	Risk of bias; inconsistency; imprecision; indirectness	Very limited (low-quality, poorly reported formula-level evidence with substantial indirectness)	Possible adjunctive benefit, but current evidence is insufficient for firm conclusions

Note: The qualitative confidence judgments in this table were informed by common evidence-appraisal considerations, including risk of bias, inconsistency, indirectness, imprecision, and potential publication bias [102,103,104]. They are intended to support narrative interpretation and should not be regarded as formal Grading of Recommendations Assessment, Development and Evaluation (GRADE) ratings. “Very limited” was assigned to exploratory, poorly controlled, biomarker-focused, or insufficiently reported evidence, whereas “relatively low” was assigned to evidence with a stronger methodological basis but downgraded for indirect attribution, small sample size, heterogeneity, inconsistency, imprecision, or limited safety reporting.

### 6.1. Direct Single-Herb and Defined-Preparation Evidence

Direct clinical evidence for *A. sinensis* remains limited. The available randomized, double-blind, placebo-controlled monotherapy trial in postmenopausal women did not demonstrate significant benefit for menopausal symptoms or estrogen-related endpoints [24]. Evidence from relatively defined preparations, such as Danggui injection, is also preliminary because reported effects are mainly based on surrogate biomarkers and incompletely reported trial methods [95]. These findings do not support broad clinical extrapolation from traditional use or preparation availability; instead, they indicate the need for preparation-specific evaluation using standardized interventions, clinically relevant endpoints, and systematic safety assessment.

### 6.2. Formula-Level Evidence and Clinical Attribution

Most clinical research involving *A. sinensis* concerns multi-herb formulas. Systematic reviews and randomized trials have reported potentially beneficial signals for Danggui Shaoyao San, Danggui Buxue Tang, Xiao Yao San, and Danggui Liuhuang Decoction in gynecological, gastrointestinal, anxiety-related, or endocrine contexts [25,96,97,98,99,100,101]. However, these findings are weakened by small or methodologically limited trials, heterogeneous formula compositions, inconsistent endpoints, and indirect attribution to *A. sinensis*. Formula-level effects, therefore, cannot establish the independent contribution of *A. sinensis* without comparative designs such as factorial, subtraction, add-on, or component-specific studies. Current clinical evidence should be regarded as hypothesis-supporting rather than confirmatory.

## 7. Phthalide Scaffold as a Translational Template: Lessons from Structure-Inspired Drug Development

Beyond traditional formulas and herbal preparations, an important translational implication of *A. sinensis* research lies in the drug-discovery potential of its phthalide constituents. Phthalides are characteristic lipophilic compounds of *A. sinensis* and related Apiaceae species and have been associated with neuroprotective, vasoprotective, and anti-inflammatory activities [105]. Emerging phthalide scaffolds may also have potential relevance to bone metabolism [106]. Although the pharmacological contribution of individual natural phthalides in whole-herb preparations remains difficult to define, their structural features provide useful templates for natural-product-inspired drug development.

Butylphthalide provides an instructive example of successful development based on a phthalide-related scaffold, although it should not be regarded as a direct clinical translation of *A. sinensis* itself. Structural analysis and optimization of phthalide-type compounds contributed to the development of dl-3-n-butylphthalide, which has been used clinically for acute ischemic stroke [107]. A multicenter randomized clinical trial registered at ClinicalTrials.gov (NCT03539445) further reported improved 90-day functional outcomes with a favorable safety profile in patients with acute ischemic stroke receiving intravenous thrombolysis and/or endovascular treatment, supporting the therapeutic potential of optimized phthalide-derived or phthalide-inspired agents [27]. This example illustrates that natural phthalide scaffolds can inform drug design even when the final agent is not directly derived from the crude herb. Recent studies on falcarinphthalide A, a phthalide identified from *A. sinensis*, further highlight the scaffold value of this constituent class. Falcarinphthalide A has been reported as a structurally novel phthalide with potential relevance to bone metabolism, and total synthesis studies have supported its accessibility for further pharmacological and medicinal chemistry investigation [106]. However, its drug-development value remains at an early stage and requires validation through target identification, structure–activity relationship analysis, pharmacokinetic evaluation, safety assessment, and in vivo efficacy studies.

Taken together, phthalides represent both bioactive constituents of *A. sinensis* and structurally informative natural products for drug discovery. The experience with butylphthalide and emerging data on falcarinphthalide A suggest that future translational research should distinguish two related but different pathways: standardization and clinical evaluation of *A. sinensis* preparations, and medicinal chemistry development of phthalide-inspired lead compounds. This distinction avoids over-attributing the clinical effects of optimized phthalide drugs to the whole herb while recognizing the scaffold value of *A. sinensis* constituents.

## 8. Toxicology and Safety Assessment of *Angelica sinensis*

*A. sinensis* is generally considered to have relatively low toxicity when used orally as a herbal preparation, but its safety profile depends on formulation type, constituent composition, dose, route of administration, and evidence quality. Reported adverse reactions are usually mild, including gastrointestinal discomfort, allergic skin reactions, and possible photosensitivity. However, available safety data remain uneven, and many studies lack systematic adverse-event monitoring, standardized preparations, or long-term follow-up.

Safety concerns vary markedly across formulations. Injectable preparations require particular caution because direct systemic exposure may increase the risk of immediate hypersensitivity and anaphylactoid reactions, which are major safety concerns for Angelica-related injections [108]. Oral decoctions, extracts, and compound formulas appear to have lower acute risk, but their safety interpretation is complicated by variation in composition, processing, dosage, and co-administered herbs. Constituent-specific assessment also remains incomplete. Phthalide-rich and volatile oil fractions require attention because Z-LIG is chemically unstable and has complex pharmacokinetic behavior. For *A. sinensis* polysaccharides, current concerns relate mainly to insufficient long-term safety data, possible immunogenicity, structural heterogeneity, and batch variability, especially for purified, modified, or delivery-system-based preparations.

Potential herb–drug interactions are another important issue. Concomitant use with anticoagulant or antiplatelet agents warrants caution because *A. sinensis* may increase bleeding risk, particularly when combined with warfarin or related drugs [109,110]. By contrast, interactions with hormone-related or metabolism-related medications remain largely speculative because direct clinical evidence is limited. Overall, the most consistent safety concerns involve injectable formulations and bleeding-related interactions, whereas many other risks remain insufficiently defined. Future safety studies should emphasize formulation-specific toxicology, standardized chemical characterization, dose–exposure assessment, systematic adverse-event reporting, long-term monitoring, and clinically relevant evaluation of herb–drug interactions [111].

## 9. Discussion: Critical Translational Challenges

The main translational challenge for *A. sinensis* is to determine which development pathway is justified by the available evidence. Current data do not support a single unified model. Instead, *A. sinensis* should be evaluated through distinct routes, including standardized herbal-preparation development and natural-product-derived lead discovery. These routes differ in material definition, quality-control requirements, pharmacological validation, regulatory logic, and clinical interpretation. Evidence supporting one route should therefore not be extrapolated uncritically to another. For example, butylphthalide supports the drug-development potential of phthalide-related scaffolds, but it does not directly validate whole *A. sinensis*, crude extracts, or traditional preparations [27]. For standardized herbal preparations, preparation-level reproducibility is essential. Future studies should define botanical source, processing conditions, extraction method, formulation, marker content, stability, and batch consistency before linking a preparation to pharmacological or clinical effects. This is particularly important because processing, extraction, storage, phthalide instability, and polysaccharide heterogeneity may substantially alter preparation identity and biological activity [112,113]. In contrast, lead-compound development requires target identification, structural optimization, exposure improvement, metabolite characterization, and conventional safety evaluation. Maintaining this distinction is necessary to avoid conflating isolated-compound evidence, extract-level activity, and formula-level clinical signals.

A second priority is to move from descriptive activity reporting to decision-oriented evidence generation. Pathway modulation or disease-model improvement alone is insufficient for translational interpretation [114]. Studies should clarify whether the tested material is chemically reproducible, whether the dose or concentration is exposure-relevant, whether the effect is attributable to *A. sinensis*, and whether the endpoint is clinically meaningful. This is especially relevant for FA-related evidence, because FA is not specific to *A. sinensis* and is widely present in foods, cereals, and other medicinal or dietary plants. Findings from purified FA or non-*A. sinensis* sources should therefore be interpreted as FA-associated support rather than direct herb-specific validation unless the source, matrix, content, and exposure context are clearly defined. Pharmacokinetic, tissue-distribution, metabolite, and pharmacodynamic data are therefore critical for distinguishing mechanistically informative findings from findings with realistic translational potential [115]. Similarly, clinical studies of multi-herb formulas may provide therapeutic signals, but they cannot determine the independent contribution of *A. sinensis* without attribution-focused designs [24,25,95,96,97,98,99,100]. Claims of multi-component synergy also require restraint. The differentiated pharmacological profiles of phthalides, FA-related phenolic acids, and polysaccharides support a plausible model of constituent-class complementarity, but not confirmed synergistic interaction. Such claims should be reserved for studies using defined combinations, add-back or subtraction designs, exposure-matched dosing, and quantitative interaction models. Formulation strategies, including cyclodextrin inclusion complexes and nanoemulsions, may improve the stability and exposure of unstable constituents such as Z-LIG [116,117]. However, improved delivery should be linked to target engagement, tissue-relevant exposure, dose–response relationships, reproducible efficacy, and safety under realistic administration conditions.

Overall, future research should prioritize translational decision-making rather than further expansion of activity catalogues. Progress will depend on defining the product, aligning mechanisms with plausible exposure, separating herbal-preparation development from lead-compound discovery, quantifying proposed constituent interactions, and testing clinically interpretable outcomes.

## 10. Future Directions: A Translational Research Roadmap

Future research on *A. sinensis* should move from broad activity screening toward chemically defined, exposure-informed, and clinically attributable evaluation.

### 10.1. Standardize Raw Materials and Preparations

Preparation-specific profiles should be established for raw herbs, extracts, essential oils, polysaccharide fractions, injections, and compound formulas. Chemical fingerprinting should quantify representative phthalides and phenolic acids, while polysaccharide characterization should include molecular weight, monosaccharide composition, uronic acid content, branching features, and relevant contaminants. Documentation of plant origin, processing, storage, and batch variation would improve reproducibility and support preparation-specific quality control [112].

### 10.2. Align Mechanistic Studies with Exposure Data

Experimental concentrations should be guided by measured plasma, tissue, local, or formulation-specific exposure. Future studies should characterize phthalide stability, metabolites, and tissue distribution; measure both free and conjugated FA; and use labeled or structurally traceable polysaccharides to investigate intestinal and systemic disposition. Integration with pharmacodynamic outcomes would help identify mechanisms that remain plausible under achievable exposure conditions [115,118].

### 10.3. Define Polysaccharide Structure–Activity Relationships

Standardized extraction and fractionation procedures should be used to generate structurally defined polysaccharide fractions. Matched functional assays should then determine how molecular weight, composition, linkage patterns, branching, and conformation relate to hematopoietic, immunological, metabolic, or microbiota-associated effects. These data would support rational candidate selection, batch-release criteria, and polysaccharide quality control [113].

### 10.4. Quantitatively Test Constituent Interactions

Interactions among phthalides, phenolic acids, and polysaccharides should be tested using chemically defined recombination, fraction-ablation, and reconstitution designs. Individual fractions and their combinations should be evaluated in matched models and analyzed using approaches such as Chou–Talalay, Bliss independence, Loewe additivity, or highest single agent [92,93,94]. This would distinguish genuine synergy or antagonism from additive or independent activity.

### 10.5. Conduct Formulation-Specific Clinical Studies

Clinical development should prioritize standardized single-herb preparations or clearly defined constituent-enriched formulations selected on the basis of preparation-specific preclinical evidence. Trials should use adequate randomization, blinding, sample sizes, validated endpoints, batch-level chemical profiles, exposure markers where feasible, and systematic safety monitoring. Designs should distinguish effects attributable to *A. sinensis* from those of multi-herb formulas or phthalide-inspired drugs and follow relevant Consolidated Standards of Reporting Trials (CONSORT) guidance for herbal interventions. Recent evaluations also indicate that reporting quality remains an important issue in Chinese herbal medicine trials [119,120,121].

## 11. Conclusions

*A. sinensis* should be evaluated through a constituent-specific and exposure-relevant translational framework rather than by aggregating heterogeneous pharmacological activities. Current evidence indicates that phthalides, FA-related phenolic acids, and polysaccharides differ substantially in stability, exposure, biological targets, and preparation dependence, and therefore should not be interpreted as interchangeable contributors to whole-herb activity.

The preclinical literature supports multiple plausible mechanisms, including regulation of inflammation, oxidative stress, cell fate, barrier integrity, metabolism, and hematopoietic microenvironment function. However, many mechanisms remain exposure-constrained or difficult to attribute because of supraphysiological in vitro concentrations, undefined preparations, polysaccharide heterogeneity, and limited pharmacokinetic/pharmacodynamic validation. Clinical evidence is also limited, as direct evidence for single-herb or chemically defined *A. sinensis* preparations remains sparse, whereas most clinical signals derive from multi-herb formulas.

Future translation should proceed through clearly separated development routes: standardized herbal preparations supported by reproducible chemical and pharmacological profiles, and phthalide-inspired lead-compound discovery supported by conventional target, exposure, efficacy, and safety validation. This distinction may help move the field from broad activity claims toward evidence that is reproducible, attributable, and clinically interpretable.

## Data Availability

No new data were created or analyzed in this study. Data sharing is not applicable to this article.

## Supplementary Materials

The following supporting information can be downloaded at: https://www.mdpi.com/article/10.3390/ph19071073/s1, Table S1: Study-level qualitative translational appraisal of pharmacological studies on Angelica sinensis constituents and preparations.
