# Supplementary material for: Angelica sinensis as a Multi-Targeted Natural Product Candidate: Constituent-Specific Mechanisms, Exposure Constraints, and Translational Development Challenges
Source: Pharmaceuticals (Basel). 2026 Jul 12;19(7):1073. doi: 10.3390/ph19071073 (PMC13415647; doi:10.3390/ph19071073)
Supplement: Supplementary file 1 [file pharmaceuticals-19-01073-s001.zip › pharmaceuticals-4368945-supplementary.pdf]

Angelica sinensis as a Multi-Targeted Natural Product Candidate: Constituent-Specific Mechanisms, Exposure Constraints, and Translational Development Challenges

Jialian Song [1], Hui Sun [1], Zhineng Li [1], Guangli Yan [1,*], Ling Kong [1], Lei Liu [1] and Xijun Wang [1,2,*]

Supplementary Table S1. Study-level qualitative translational appraisal of pharmacological studies on *Angelica sinensis* constituents and preparations.

Category	Study	Component	Model	Dose/concentration	Mechanistic support	Exposure plausibility	Translational interpretation	Main mechanism/effect
Neuroprotection	[57]	Angelica sinensis polysaccharide(s)	amyloid beta 25–35-induced Alzheimer's disease rats	50 mg/kg	Strong: in vivo model with K252a-based pathway inhibition	Not assessable for classical pharmacokinetics; in vivo dosing supports biological relevance	Moderate–strong; mechanistically supported but preparation-specific validation is needed	brain-derived neurotrophic factor/tropomyosin receptor kinase B/cyclic adenosine monophosphate response element-binding protein pathway
Neuroprotection	[58]	Z-ligustilide	hydrogen peroxide-injured pheochromocytoma 12 cells	0.1–5 μg/mL	Moderate: cell model with oxidative injury endpoints	Limited to marginal; upper range may exceed reported oral systemic exposure after unit conversion	Moderate–low; useful for target exploration but requires exposure-matched or in vivo confirmation	Antioxidant effect and inhibition of mitochondrial apoptosis
Neuroprotection	[18]	Ligustilide, isomer not specified	oxygen-glucose deprivation/reoxygenation-injured pheochromocytoma 12 cells	10^-6 mol/L	Strong: supported by 3-methyladenine and dorsomorphin inhibition	Plausible to marginal; low micromolar exposure is closer to reported phthalide exposure than high-dose models	Moderate–strong; mechanistically informative, but parent/metabolite exposure should be confirmed	adenosine monophosphate-activated protein kinase/mammalian target of rapamycin/autophagy regulation; anti-apoptosis

Category	Study	Component	Model	Dose/concentration	Mechanistic support	Exposure plausibility	Translational interpretation	Main mechanism/effect
Neuroprotection	[61]	Angelica sinensis extract	Global cerebral ischemia	0.25/0.5/1 g/kg	Strong: in vivo validation with SB203580 blockade	Not assessable; crude extract composition and constituent exposure are preparation-dependent	Moderate–strong, formulation-specific; effects cannot be assigned to a single constituent	p38 mitogen-activated protein kinase-related anti-neuronal apoptosis
Neuroprotection	[62]	Angelica sinensis extract	Brain ischemia with neurogenesis focus	0.25/0.5/1 g/kg	Strong: in vivo evidence with pathway-level validation	Not assessable; extract-level evidence with uncertain constituent attribution	Moderate, formulation-specific; supports extract-level neuroprotection but requires constituent attribution	cyclic adenosine monophosphate response element-binding protein-brain-derived neurotrophic factor-related neurogenesis
Neuroprotection	[19]	Ferulic acid	chronic constriction injury rats plus Schwann cell and microglia models	100 mg/kg; 2 μM	Moderate–strong: ribonucleic acid sequencing and inhibitor-supported validation	Marginal; 2 μM exceeds reported free ferulic acid exposure but is closer than high-micromolar models	Moderate–strong; relatively exposure-aware evidence, but free and conjugated ferulic acid should be measured	toll-like receptor 4/myeloid differentiation primary response 88/nuclear factor kappa B inhibition; anti-inflammatory and pro-repair effects

Category	Study	Component	Model	Dose/concentration	Mechanistic support	Exposure plausibility	Translational interpretation	Main mechanism/effect
Neuroprotection	[59]	Ligustilide, isomer not specified	spinal cord injury mice plus tert-butyl hydroperoxide-injured pheochromocytoma 12 cells	20/50 mg/kg; 20/50 μM	Strong: in vivo and in vitro validation with small interfering ribonucleic acid targeting BCL2-interacting protein 3	Limited for the in vitro arm; 20–50 μM likely exceeds conventional oral systemic exposure	Moderate; mechanistically strong but exposure-constrained	BCL2-interacting protein 3-mediated mitophagy activation; anti-apoptosis
Neuroprotection	[60]	Z-ligustilide	intervertebral disc degeneration plus nucleus pulposus cell pyroptosis	10/20/40 μM; 10/20 mg/kg/day	Strong: in vivo and in vitro validation with small interfering ribonucleic acid targeting autophagy-related protein 5	Limited; 10–40 μM exceeds reported oral systemic phthalide exposure, although local tissue relevance is possible	Moderate; mechanistically strong but requires tissue-specific pharmacokinetic validation	autophagy-related protein 5/NOD-like receptor family pyrin domain-containing 3 regulation; anti-pyroptosis
Metabolic regulation	[63]	Angelica sinensis polysaccharide(s)	KKAy diabetic mice	400 mg/kg/day for 4 weeks	Moderate–low: in vivo multi-omics association without direct blockade	Not assessable for classical pharmacokinetics; gut-associated exposure may be relevant	Moderate–low; hypothesis-generating evidence for Angelica sinensis polysaccharide-mediated metabolic regulation	Gut microbiota remodeling; glucose improvement

Category	Study	Component	Model	Dose/concentration	Mechanistic support	Exposure plausibility	Translational interpretation	Main mechanism/effect
Metabolic regulation	[65]	Ferulic acid	high-fat diet-induced non-alcoholic fatty liver disease mice	100 mg/kg/day for 12 weeks	Moderate–strong: in vivo model with pathway validation	Dose-based in vivo evidence; free ferulic acid systemic exposure remains uncertain	Moderate; relevant in vivo evidence, but pharmacokinetic/pharmacodynamic linkage is needed	β-oxidation activation; anti-steatosis
Metabolic regulation	[64]	Angelica sinensis polysaccharide(s)	high-fat diet-induced metabolic disorder plus insulin resistance-HepG2 cells	80–320 mg/kg/day	Moderate–strong: LY294002-supported pathway involvement	Not assessable for classical pharmacokinetics; in vivo dosing supports biological relevance	Moderate; promising preclinical evidence but preparation-dependent	peroxisome proliferator-activated receptor gamma/sirtuin 1-adenosine monophosphate-activated protein kinase regulation; improved lipid metabolism and insulin sensitivity
Metabolic regulation	[66]	Ferulic acid	palmitate-induced HepG2 metabolic stress	50/100/200 μM	Moderate–low: cell model only	Limited; high-micromolar concentrations likely exceed free ferulic acid exposure	Low; mainly hypothesis-generating unless validated at exposure-compatible concentrations	adenosine monophosphate-activated protein kinase activation and mitochondrial homeostasis

Category	Study	Component	Model	Dose/concentration	Mechanistic support	Exposure plausibility	Translational interpretation	Main mechanism/effect
Metabolic regulation	[67]	Ferulic acid	endurance model plus C2C12 myotubes	0.5% diet; 0.5 μM	Moderate–strong: ML385 inhibition supports nuclear factor erythroid 2-related factor 2 involvement	Plausible; 0.5 μM is close to reported free ferulic acid exposure	Moderate–strong; one of the more exposure-compatible ferulic acid mechanistic studies	adenosine monophosphate-activated protein kinase/peroxisome proliferator-activated receptor gamma coactivator 1-alpha/nuclear factor erythroid 2-related factor 2 activation; improved endurance
Intestinal inflammation and barrier injury	[20]	Ferulic acid	radiation-induced intestinal injury	10/30/90 mg/kg	Moderate: in vivo protection with limited causal validation	Dose-based in vivo evidence; local intestinal exposure may be relevant	Moderate; biologically plausible for intestinal protection, but local ferulic acid exposure should be measured	Anti-inflammatory and antioxidant intestinal protection
Intestinal inflammation and barrier injury	[68]	Ferulic acid	lipopolysaccharide-induced Caco-2 barrier dysfunction	25/50/100 μM	Moderate–strong: pathway-supported barrier restoration	Limited for systemic exposure; local intestinal exposure may be possible but unconfirmed	Moderate; relevant to epithelial barrier models but not yet exposure-validated	phosphoinositide 3-kinase/protein kinase B-related barrier restoration

Category	Study	Component	Model	Dose/concentration	Mechanistic support	Exposure plausibility	Translational interpretation	Main mechanism/effect
Intestinal inflammation and barrier injury	[69]	Ferulic acid	sodium arsenite-induced colon injury	100 mg/kg; 40 μM	Moderate: in vivo and in vitro evidence without direct blockade	Marginal to limited; 40 μM exceeds free systemic exposure, but local intestinal relevance is possible	Moderate–low; requires local tissue concentration or pharmacokinetic/pharmacodynamic validation	Tight junction protection; anti-inflammatory effect
Intestinal inflammation and barrier injury	[70]	ginsenoside Rh2/lipoic acid-modified ultrasonic Angelica sinensis polysaccharide nanoparticles	dextran sulfate sodium-induced colitis	approximately 10 mg/kg	Moderate–strong: targeted delivery with in vivo validation	Formulation-specific; colon-targeted delivery may generate local exposure	Moderate, formulation-specific; promising but not generalizable to ordinary Angelica sinensis polysaccharide preparations	Colon-targeted delivery; microbiota and short-chain fatty acid modulation
Intestinal inflammation and barrier injury	[71]	Angelica sinensis aboveground-part polysaccharide	dextran sulfate sodium-induced colitis plus IPEC-J2 cells	50/100/150 mg/kg; 5–20 μg/mL	Moderate–strong: in vivo and in vitro evidence with pathway support	Part-specific polysaccharide preparation; mainly relevant to local intestinal and gut-associated effects	Moderate, formulation-specific; not directly generalizable to unmodified Angelica sinensis polysaccharide(s)	toll-like receptor 4/myeloid differentiation primary response 88/nuclear factor kappa B inhibition; barrier improvement
Intestinal inflammation and barrier injury	[73]	Angelica oil	dextran sulfate sodium-induced colitis	10/20/40 mg/kg	Moderate–low: omics association without direct causal validation	Not assessable; Angelica oil composition and constituent exposure are variable	Moderate–low, formulation-dependent; mainly association-based evidence	Microbiota and sphingolipid metabolism regulation

Category	Study	Component	Model	Dose/concentration	Mechanistic support	Exposure plausibility	Translational interpretation	Main mechanism/effect
Intestinal inflammation and barrier injury	[72]	Angelica sinensis polysaccharide(s)	alcoholic fatty liver disease-associated gastrointestinal injury	100 mg/kg	Moderate: in vivo evidence without direct blockade	Not assessable for classical pharmacokinetics; gut-associated mechanisms are plausible	Moderate; supports Angelica sinensis polysaccharide-related gut protection but requires structural characterization	short-chain fatty acid-related utilization and barrier homeostasis
Hematopoietic microenvironment regulation	[74]	Angelica sinensis polysaccharide(s)	5-fluorouracil-injured HS-5 stromal cells plus co-culture	100 µg/mL	Moderate: cell and co-culture functional evidence	Not assessable; in vitro concentration lacks direct exposure linkage	Moderate–low; useful for niche-mechanism exploration but requires in vivo confirmation	Preserves hematopoietic-supportive niche
Hematopoietic microenvironment regulation	[75]	Angelica sinensis polysaccharide(s)	D-galactose-induced aging hematopoietic stem and progenitor cells	200 mg/kg/day	Moderate: in vivo functional evidence	Not assessable for classical pharmacokinetics; in vivo dosing supports biological relevance	Moderate; relevant to hematopoietic aging, but preparation definition is needed	Delays hematopoietic stem and progenitor cell senescence
Hematopoietic microenvironment regulation	[76]	Angelica polysaccharide iron complex	iron-deficiency anemia rats	12.5/25/50 mg iron/kg	Low: mainly phenotype-based evidence	Formulation-specific; iron complex exposure differs from crude Angelica sinensis polysaccharide(s)	Low–moderate, formulation-specific; supports hematological improvement but has limited mechanistic depth	Improves hematological parameters

Category	Study	Component	Model	Dose/concentration	Mechanistic support	Exposure plausibility	Translational interpretation	Main mechanism/effect
Hematopoietic microenvironment regulation	[77]	Angelica sinensis polysaccharide(s)	pregnancy-related iron-deficiency anemia	200/400 mg/kg	Moderate: in vivo evidence with pathway support	Not assessable for classical pharmacokinetics; in vivo dosing supports biological relevance	Moderate; plausible hematopoietic evidence requiring standardized Angelica sinensis polysaccharide validation	Hepcidin-ferroportin 1 regulation; promotes erythropoiesis
Hematopoietic microenvironment regulation	[78]	Angelica sinensis polysaccharide(s)	chronic kidney disease-related anemia	0.5/1 g/kg	Moderate: in vivo evidence with pathway support	Not assessable for classical pharmacokinetics; relatively high in vivo dose	Moderate; supports renal-anemia relevance but needs pharmacokinetic/pharmacodynamic-compatible biomarkers	hypoxia-inducible factor 2 alpha/erythropoietin regulation; improves iron availability
Others	[79]	Angelica sinensis polysaccharide(s)	oxygen-glucose deprivation-injured H9c2 cells and rat acute myocardial infarction model	50 µg/mL; 100/200/400 mg/kg for 4 weeks	Strong: activating transcription factor 6 silencing and adenosine monophosphate-activated protein kinase modulation support causality	Not assessable for classical pharmacokinetics; in vivo arm supports functional relevance	Moderate–strong; mechanistically strong but preparation-specific	activating transcription factor 6/adenosine monophosphate-activated protein kinase regulation; cardioprotection

Category	Study	Component	Model	Dose/concentration	Mechanistic support	Exposure plausibility	Translational interpretation	Main mechanism/effect
Others	[21]	Angelica sinensis polysaccharide(s)	sodium nitroprusside-induced osteoarthritis chondrocytes	50/200 μg/mL	Moderate–strong: chloroquine, 3-methyladenine, and SCH772984 support autophagy involvement	Not assessable; cell-only Angelica sinensis polysaccharide exposure lacks in vivo linkage	Moderate–low; mechanistically informative but requires in vivo validation	extracellular signal-regulated kinase 1/2-autophagy regulation; anti-apoptosis
Others	[84]	Ferulic acid	CT26 cells plus tumor-bearing mice	0–800 μM; 20/40/80 mg/kg	Moderate–low: limited mechanistic validation	Limited for the in vitro arm; 0–800 μM is largely supraphysiological relative to free ferulic acid exposure	Low; mainly hypothesis-generating for ferulic acid-related antitumor activity	mitogen-activated protein kinase/autophagy-associated apoptosis
Others	[89]	N-butylidenephthalide	breast cancer cells	12.5–100 μg/mL	Moderate–strong: caspase inhibitor rescue supports apoptosis mechanism	Unclear; N-butylidenephthalide exposure from Angelica sinensis preparations is not established	Low–moderate, compound-specific; not directly attributable to whole Angelica sinensis preparations	Caspase-dependent apoptosis and radiosensitization

Category	Study	Component	Model	Dose/concentration	Mechanistic support	Exposure plausibility	Translational interpretation	Main mechanism/effect
Others	[80]	Angelica sinensis polysaccharide(s)	lipopolysaccharide-induced primary dairy cow claw dermal cells	1–100 μg/mL	Moderate–low: cell model only	Not assessable; lacks in vivo exposure linkage	Low–moderate; preliminary anti-inflammatory evidence	nuclear factor kappa B/mitogen-activated protein kinase inhibition; anti-inflammatory effect
Others	[81]	Angelica sinensis polysaccharide(s)	5-fluorouracil-induced liver injury	100 mg/kg; 100 μg/mL	Moderate–strong: in vivo and in vitro evidence with nuclear factor erythroid 2-related factor 2 pathway support	Not assessable for classical pharmacokinetics; in vivo arm supports biological relevance	Moderate; relevant hepatoprotective evidence requiring standardized Angelica sinensis polysaccharide characterization	nuclear factor erythroid 2-related factor 2 activation; hepatoprotection
Others	[85]	Ferulic acid	diabetic nephropathy mice	200 mg/kg/day	Moderate–low: limited causal validation	Dose-based in vivo evidence; free and conjugated ferulic acid exposure is uncertain	Moderate–low; plausible but not exposure-validated	autophagy/NOD-like receptor family pyrin domain-containing 3 regulation; renoprotection
Others	[22]	Ligustilide, isomer not specified	tumor necrosis factor-alpha-treated human umbilical vein endothelial cells	1–10 μM	Moderate: cell model only	Marginal; lower range is closer to reported phthalide exposure, whereas upper range may exceed systemic exposure	Moderate–low; requires in vivo vascular exposure validation	nuclear factor erythroid 2-related factor 2/heme oxygenase 1 activation; anti-vascular inflammation

Category	Study	Component	Model	Dose/concentration	Mechanistic support	Exposure plausibility	Translational interpretation	Main mechanism/effect
Others	[90]	Z-ligustilide	ultraviolet B-injured keratinocytes	10 μM	Strong: small interfering ribonucleic acid targeting nuclear factor erythroid 2-related factor 2 and tin protoporphyrin IX support pathway involvement	Route-specific; 10 μM exceeds reported oral plasma peak but may be relevant for topical or local exposure	Moderate, route-specific; mechanistically strong but exposure route should be specified	nuclear factor erythroid 2-related factor 2/heme oxygenase 1 activation and nuclear factor kappa B inhibition; cytoprotection
Others	[82]	Angelica sinensis polysaccharide(s)	carbon tetrachloride-induced liver fibrosis	200 mg/kg/day	Strong: interleukin-22/signal transducer and activator of transcription 3 blockade supports causality	Not assessable for classical pharmacokinetics; in vivo dosing supports biological relevance	Moderate–strong; strong preclinical evidence but preparation-dependent	interleukin-22/signal transducer and activator of transcription 3 regulation; anti-fibrotic effect
Others	[86]	Ferulic acid	lipopolysaccharide-induced pneumonia plus macrophages	25/50/100 mg/kg; 10/100 μM	Strong: chemical proteomics and target validation	Limited for the in vitro arm; 10–100 μM exceeds reported free ferulic acid systemic exposure	Moderate; mechanistically strong but exposure-constrained	peroxiredoxin 1-toll-like receptor 4-nuclear factor kappa B inhibition; anti-inflammatory effect

Category	Study	Component	Model	Dose/concentration	Mechanistic support	Exposure plausibility	Translational interpretation	Main mechanism/effect
Others	[87]	Ferulic acid	radiation-induced liver injury	50 mg/kg	Moderate: in vivo evidence only	Dose-based in vivo evidence; free and conjugated ferulic acid exposure not defined	Moderate–low; biologically plausible but mechanism and pharmacokinetic/pharmacodynamic linkage need strengthening	Janus kinase/signal transducer and activator of transcription-nuclear factor erythroid 2-related factor 2 regulation; hepatoprotection
Others	[91]	Ligustilide, isomer not specified	osteoarthritis model with cells and rats	25/50 µM; intra-articular 75/150 µM	Strong: in vivo and in vitro mechanistic evidence	Route-specific; oral systemic exposure is implausible, but intra-articular exposure may be locally relevant	Moderate, route-specific; not directly applicable to oral Angelica sinensis preparations	c-Jun N-terminal kinase/p38 mitogen-activated protein kinase inhibition; anti-cartilage degeneration
Others	[83]	Angelica sinensis polysaccharide(s)	U251 glioma cells plus xenograft	0.2 mg/mL; 50 mg/kg	Moderate–low: in vivo antitumor effect with limited mechanism depth	Not assessable for classical pharmacokinetics; high in vitro macromolecular concentration lacks exposure linkage	Low–moderate; preliminary and disease-specific evidence	Anti-glioma activity

Category	Study	Component	Model	Dose/concentration	Mechanistic support	Exposure plausibility	Translational interpretation	Main mechanism/effect
Others	[23]	Angelica sinensis polysaccharide(s) plus cisplatin	cisplatin-resistant ovarian cancer	Angelica sinensis polysaccharide(s) 200 µg/mL; Angelica sinensis polysaccharide(s) 0.2 mg/kg/day	Strong: glutathione peroxidase 4 rescue and ferrostatin-1 support ferroptosis mechanism	Combination-specific; Angelica sinensis polysaccharide exposure and dose–response linkage remain unclear	Moderate; mechanistically strong but combination-specific	glutathione peroxidase 4-dependent ferroptosis; enhanced cisplatin sensitivity
Others	[88]	Ferulic acid	septic acute lung injury plus lipopolysaccharide-injured cells	100 mg/kg; 0.1 µM	Strong: short hairpin ribonucleic acid targeting nuclear factor erythroid 2-related factor 2 and ferric citrate rescue support causality	Plausible for the in vitro concentration; 0.1 µM is below reported free ferulic acid systemic exposure, although lung tissue exposure remains unmeasured	Moderate–strong; mechanistically supported and exposure-compatible at the cellular concentration, but tissue pharmacokinetic/pharmacodynamic validation is still needed	nuclear factor erythroid 2-related factor 2/heme oxygenase 1-mediated anti-ferroptosis and barrier protection
